# A Retrospective Observational Study of Quality of Life in a Northern Greece Population of People with Haemophilia

**DOI:** 10.3390/life14060697

**Published:** 2024-05-29

**Authors:** Eleni Moka, Zacharo Ntova, Eleni Gavriilaki, Nikolaos Kotsiou, Sofia Chissan, Theodosia Papadopoulou, Sofia Vakalopoulou

**Affiliations:** 2nd Propedeutic Department of Internal Medicine, Hippokration General Hospital, 54642 Thessaloniki, Greece; elenimoka30@gmail.com (E.M.); ntovairo@gmail.com (Z.N.); kotsiounikolaos@gmail.com (N.K.); sofiachissan@yahoo.com (S.C.); sissipapth@yahoo.gr (T.P.); svakalopoulou@yahoo.com (S.V.)

**Keywords:** haemophilia, health-related quality of life, Greece, Haem-A-QoL, age, prophylaxis

## Abstract

Haemophilia presents a significant challenge to the quality of life of affected individuals. Evaluating the health-related quality of life (HRQoL) of people with haemophilia (PwH) provides a valuable mean of assessing their perception of overall care outcomes, while also identifying influential factors across various age and condition severity demographics. This observational retrospective study determined the HRQoL of 100 adult PwH in Northern Greece through comprehensive analysis and interpretation of their HRQoL levels, particularly in domains concerning their physical, emotional, and mental well-being, obtained through the Haem-A-QoL index questionnaire. Disease severity and young age were significantly associated with the administration of prophylactic treatment (84.2% of patients with severe haemophilia and 65.2% of patients aged 18–30). The mean Haem-A-QoL score was 40.11 ± 17.38, with the lowest HRQoL observed in the 46–60 age group (46.16), and the highest in the ≥61 age groups (35.16). Notably, the ‘Sports/Leisure’ and ‘Physical Health’ domains exhibited the highest scores, in contrast to ‘Family Planning’ and ‘Relationships/Sexuality’. Individuals with mild haemophilia recorded the lowest mean score (39.38), while those with a severe condition exhibited the highest (41.23). Age, disease severity, and physical activity emerged as primary determinants significantly affecting HRQoL outcomes.

## 1. Introduction

Haemophilia, an X-linked bleeding disorder primarily affecting males, results from an inherited deficiency of either coagulation factor VIII (haemophilia A) or factor IX (haemophilia B) [1]. The severity of haemophilia correlates with the risk of bleeding, depends on the factor levels in functional plasma, and is categorized as mild (5–40%), moderate (1–5%) or severe (<1%) [2]. The most common manifestations of the condition are recurrent bleeding episodes, primarily affecting the joints and muscles, that may lead to various long-term clinical implications such as restricted range of motion, musculoskeletal disorders, and chronic joint disease [3,4,5]. Furthermore, challenges and limitations such as frequent hospital visits, regular treatment injections, absenteeism from school or work, and restricted participation in activities, including sports, are commonly experienced [6,7,8,9]. Consequently, both haemophilia and its treatment have a considerable impact on patients’ health-related quality of life (HRQoL) and daily functioning.

While its precise definition may elude a singular interpretation, the World Health Organization (WHO) defines quality of life as “an individual’s perception of their position in life in the context of the culture and value systems in which they live and in relation to their goals, expectations, standards and concerns” [10]. The term HRQoL was based on the WHO definition and encompasses various dimensions of well-being and functionality, including physical, emotional, mental, social, and behavioral aspects, as perceived by both patients and observers [11,12]. HRQoL is shaped by the presence of a disease, its treatment, and by personal attributes, such as coping mechanisms or locus of control, socioeconomic status, and living conditions [6,13]. Research involving people with chronic conditions indicates that such patients frequently prioritize interpersonal connections and altruistic activities over their own health and professional ambitions. Many of these individuals express satisfaction with their life achievements despite their health challenges. Clinicians find interest in understanding these sentiments and the communication outcomes among patients with chronic diseases, as they seek to evaluate their patients’ overall HRQoL [14].

Evaluating HRQoL not only provides a reliable measurement of patients’ perceptions regarding the overall effectiveness of care but also is crucial in appraising both existing and novel treatment strategies [15,16,17,18,19]. In addition, HRQoL assessments enable cross-country comparisons of healthcare systems, potentially serving as an important tool to exert pressure on health authorities to align national health services with those of countries offering superior care standards [20]. Finally, the correct evaluation of the patient’s answers to the HRQoL questionnaires can contribute to the early diagnosis of problems that the individual does not report, such as depression and sexual dysfunction [21,22,23,24]. 

The aim of this investigation was to evaluate the HRQoL among people with haemophilia (PwH) within the Haemophilia Center of Northern Greece. Our study population consisted of 100 adult PwH and was representative of the demographic diversity observed in this region. By comprehensively analyzing HRQoL levels across various domains, such as physical, emotional, and mental well-being of the individuals, we aimed to provide insights into how different demographic and clinical factors (i.e., age, severity of haemophilia, administration of prophylactic treatment) influence the perceived HRQoL of PwH. 

## 2. Materials and Methods

### 2.1. Study Population

Consecutive PwH enrolled and monitored at the Haemophilia Center of Northern Greece were included in the study. Individuals with factor levels below 1% were classified as having severe hemophilia, while those with levels between 1% and 5% were categorized as moderate, and those with levels exceeding 5% were classified as mild. The sole inclusion criteria were the ability to comprehend the questions, consent to complete the questionnaire, and being aged 18 years or older. The questionnaire administration occurred between March 2019 and November 2019 at our center, with patients completing the questionnaire in person. Participation in the study was voluntary, and the questionnaires did not elicit identifiable information or details about the patients’ medical condition [25]. The study was conducted in accordance with the Declaration of Helsinki and approved by the Institutional Review Board of Hippokration General Hospital. Informed consent was obtained from all subjects involved in the study.

### 2.2. Measuring Tool

The Haem-A-QoL quality of life index questionnaire for adult with haemophilia was utilized for our research. The questionnaire has undergone thorough evaluation across multiple studies to assess its reliability, validity, and specificity. Haem-A-QoL comprises 46 items organized into 10 dimensions, including physical health, feelings, self-awareness, leisure and sports, work and school, dealing with the condition, treatment satisfaction, future prospects, and family planning and partnership-sexuality, along with a cumulative score scale [26]. Scoring involves transforming the attained scores for each dimension, as well as the overall score, onto a scale ranging from 0 to 100, where 0 denotes the optimal and 100 represents the poorest HRQoL [27]. The Greek version of the Haem-A-QoL questionnaire was used in our study after the expressed permission of the Haemo-QoL group [28].

### 2.3. Statistical Analysis

In the descriptive analysis of quantitative variables, the mean value and standard deviation (mean ± SD) were employed, while absolute value and relative frequency (*n*, %) were used for qualitative variables.

For variables with a number of subjects <50, the Shapiro–Wilk normality test was used, and the Kolmogorov–Smirnov test was utilized for the variables exceeding this threshold.

To establish correlations between the scores of the 10 subscales and the total score of the Haem-A-QoL questionnaire with disease severity and age groups, tests were conducted to compare quantitative variables among three or more independent groups. The parametric analysis of variance (One Way ANOVA) was employed if subscale scores followed a normal distribution; otherwise, the non-parametric Kruskal–Wallis test was used.

Similarly, to correlate the scores of the 10 subscales and the total score of the Haem-A-QoL questionnaire with prophylactic treatment, tests were conducted to compare quantitative variables among two independent groups. The parametric Independent Samples *t*-test was applied if subscale scores followed a normal distribution, while the non-parametric Mann–Whitney test was used otherwise.

The χ^2^ test was employed to examine correlations between demographic and clinical characteristics. For data analysis, the statistical software “IBM SPSS Statistics, Version 20.0” was utilized, with a significance level set at 5%.

## 3. Results

In this study, a cohort of 100 consecutive adult individuals diagnosed with haemophilia A or B participated by completing the Haem-a-QoL questionnaire. All participants were male, aged from 18 to 76 years, and exhibited haemophilia severity ranging from mild to severe. The sampled population represented diverse geographical areas, encompassing both the urban and rural regions of Macedonia, Thrace, Thessaly, and the islands of the North Aegean. The detailed demographic and clinical characteristics of the sample population are provided in Table 1.

A notable correlation was observed between disease severity and the prophylactic treatment (Table 2). Specifically, the proportion of patients with severe haemophilia under prophylaxis was significantly higher (84.2%) compared to individuals with moderate (22.2%) and mild (2.3%) haemophilia.

Furthermore, a statistically significant correlation was observed between disease severity and age groups, with the highest percentages of patients diagnosed with severe haemophilia found within the age brackets of 18–30 years and 31–45 years, accounting for 65.5% and 45.2%, respectively. Additionally, there was a statistically significant association between prophylaxis and age groups, as evidenced by a higher likelihood of patients aged 18–30 receiving prophylactic treatment compared to those in other age categories (Table 3).

Table 4 displays the descriptive attributes (mean value, standard deviation, frequencies, and percentages) of responses to each questionnaire within the study cohort. The overall mean Haem-A-QoL score among all participants was 40.11 ± 17.38. Across the 10 subscales, the mean score ranged from 15.68 to 58.45.

Table 5 presents the statistical parameters (mean value, standard deviation, and minimum and maximum values) of the scores across each of the 10 questionnaire subscales, categorized by age group. Additionally, it encompasses the total score of the questionnaire. Notably, the 46–60 age group exhibited the highest overall score, indicating a lower HRQoL.

Across all age groups, the domains ‘Sports/Leisure’ and ‘Physical Health’ exhibited the highest mean scores, suggesting a lower HRQoL. In contrast, domains such as ‘Family Planning’ and ‘Relationships/Sexuality’ displayed the lowest mean scores, indicative of a higher HRQoL. The lowest mean total Haem-A-QoL score across all four age groups was observed in individuals aged ≥61 years (35.16), while the highest was noted in the 46–60 age group (46.16).

Table 6 encompasses the statistical parameters (mean value, standard deviation, and minimum and maximum values) across each of the 10 questionnaire subscales, categorized by condition severity, alongside the total score.

No difference was observed among the three groups concerning the domains reflecting the lowest HRQoL(“Sports/Leisure” and “Physical Health”). Similarly, “Family Planning” and “Relationships/Sexuality” exhibited comparable lowest mean scores across different age cohorts.

In the comprehensive assessment of Haem-A-QoL scores across the three disease severities, the mildest form of the condition displayed the lowest mean value (39.38), contrasting with the highest mean value recorded in the severe manifestation (41.23).

Table 7 provides the statistical attributes (mean value, standard deviation, and minimum and maximum values) across each of the 10 questionnaire subscales for patients undergoing prophylactic treatment, alongside the total score of the questionnaire.

There was no variance observed in the outcomes concerning the domains where the highest and lowest scores were noted regarding either severity or age groups.

## 4. Discussion

This study represents the first investigation conducted at the Haemophilia Center of Northern Greece and the third in the country, with the objective of enhancing comprehension regarding the factors influencing the HRQoL of PwH. The findings shed light on the complex interplay between haemophilia and HRQoL, focusing on physical, social, and emotional dimensions.

Most patients fell within the 18–45 age range, with only a small proportion (13%) exceeding 60 years of age, with no representation of severe haemophilia within this cohort. Across all patients, irrespective of disease severity, age, or receipt of prophylaxis, and despite the improvements regarding education and self-administration techniques, the domains experiencing the lowest HRQoL were ‘Sports/Leisure’ and ‘Physical Health’, while ‘Family Planning’ and ‘Relationships/Sexuality’ consistently emerged with the highest HRQoL scores. These findings indicate that the factors mentioned may not significantly influence the PwH’s self-assessment of their health status, even though the ≥61 age group, devoid of individuals with severe haemophilia, achieved the highest overall score.

Regarding the overall comparative Haem-A-QoL scores across the three disease severities, patients with mild and moderate haemophilia exhibited a superior HRQoL compared to those with severe form, aligning with expectations. Moreover, an analysis of the total Haem-A-QoL scores across the four age groups revealed that the highest scores were recorded among patients aged over 60 years and those aged 18–30 years, while the lowest were observed in the 46–60 age group. This difference is probably explained by the fact that all patients over 60 years of age suffered from mild haemophilia and that the systematic treatment approach, particularly primary prophylactic therapy, adopted by the younger patients led to a mitigation of haemophilic arthropathy severity and thereby enhanced HRQoL. These observations align with the existing literature, which supports the conclusion that young patients diagnosed with severe haemophilia who receive early prophylactic treatment exhibit an enhanced HRQoL [29,30,31]. On the contrary, inadequate treatment or an absence of treatment during childhood and adolescence in patients aged 46–60 years resulted in severe haemophilic arthropathy, significantly impairing HRQoL due to mobility restrictions, reduced physical activity, and less autonomy. These constraints, combined with chronic pain, may cause feelings such as irritability, anger, and helplessness [32]. Simultaneously, frequent regular visits to healthcare facilities and prolonged periods of absence from work contribute to issues with unemployment. These circumstances significantly impact the individual’s self-confidence and mental health [33].

In terms of the ‘Mental’ dimension, all individuals, regardless of the severity or prophylactic treatment, exhibited a moderate HRQoL (mean score 46.38), with exceptions noted among younger individuals aged 18–30 years, who reported a moderate to good HRQoL (mean score 38.36). Similarly, in the ‘Perception’ domain, a moderate HRQoL was evident (mean score 44.00), regardless of disease severity or prophylactic treatment. However, an examination across age groups revealed a trend of younger adult patients reporting a good to moderate HRQoL(mean score 41.55), which, surprisingly, improved among patients aged over 60 years (mean score 35.38), potentially attributed to the lower disease burden of mild haemophilia among this age group.

Although 38% of PwH reported never feeling jealous of healthy individuals, several participants admitted to experiencing jealousy at varying frequencies. This could be attributed to the absence of specialized psychological support during childhood and adolescence, which may have hindered their ability to manage negative emotions, cease comparisons with healthy individuals, and recognize their uniqueness and significance. Despite encountering challenges, participants in our study reported having a relatively satisfactory sex life. The mean score (24.16) suggests a generally good HRQoL in this dimension, irrespective of age, disease severity, or prophylactic treatment. Similar findings were reported in a recent study, where participants with haemophilia expressed satisfaction with their sexual lives, perceiving no distinctive difference from healthy individuals [34].

In comparison to a previous study in Greece utilizing the Haem-A-QoL questionnaire [35], our study recorded higher scores in six out of 10 categories, indicating a poorer HRQoL in domains such as Mental Dimension, Therapy, Physical Health, Future, Perception, and Work/Study. On the contrary, domains including Family Planning, Sports/Leisure, Coping with the disease, and Relationships/Sexuality exhibited better HRQoL. The overall Haem-A-QoL score (40.11) in our study population suggests a lower overall HRQoL. No significant demographic variations were observed, except for geographic location, with the majority of the population originating from Southern Greece. The underlying causes of these differences remain uncertain and call for additional investigation, whether they arise from variations in treatment protocols, access to information, or broader social and professional environments.

Comparing our findings with another Greek study examining the impact of arthropathy on the HRQoL of PwH, our results suggest a similarly substantial effect of joint status on patient HRQoL [36]. Furthermore, comparisons with two other studies revealed notable differences: While a study by Mackensen et al. indicated a better HRQoL across all domains for PwH in Germany [26], results similar to ours were observed in the study by Mercan et al., which found a more impaired HRQoL in Turkish patients in domains related to physical health, feeling, view, school, sport, and treatment [37]. In full concordance with our results, the findings of two Brazilian studies involving 17 and 39 adult PwH showed a greater HRQoL in Family Planning and a lower HRQoL in Sports/Leisure, while the total HRQoL scores were relatively lower (36.2 and 35.55, respectively) than ours [38,39]. An investigation involving 505 Korean PwH in the adult group revealed that the dimension with the highest mean score, indicating poor HRQoL, was Sports/Leisure (62.43), followed by Self-Awareness (52.30), and Family Planning (51.19). Relationships/Sexuality was the least impaired dimension among adults (21.15), while the total questionnaire score was 38.43 [40]. Another study in India [41] exhibited an overall score of 45.92, with the most impaired domains being Physical Health (65.00) and Sports/Leisure (60.11), while the least impaired were Dealing with the Condition (24.90) and Relationship/Sexuality (32.31). Similar results were also obtained from an Iranian study of 103 PwH; however, that study had a total score that was significantly higher (51.07) than ours [42]. Last but not least, in a global multicenter non-interventional study involving 80 adult patients across 20 centers and 10 countries from Europe, Asia, Oceania, and North America (NIS; NCT02476942), participants receiving on-demand therapy demonstrated a mean Haem-A-QoL Total Score of 40.1, while participants receiving prophylactic treatment had a mean Total Score of 26.6. The Physical Health scores were 50.1 and 49.3 for individuals under on-demand treatment and 27.9 for those receiving prophylaxis. ‘Sports/Leisure’ and ‘Future Prospects’ were the most impacted domains for both groups [43]. Our study has several limitations, including an imbalanced proportion of individuals receiving prophylaxis versus on-demand therapy and the absence of half-life prolonging agents in the treatment protocol at the time of the study. Another constraint of our research lies in the potential influence of comorbidities on the observed HRQoL. In this investigation, only the documentation of comorbid conditions was undertaken, without discerning their specific impact. While the sample size of 100 individuals is deemed representative for Northern Greece, further inquiry with a larger cohort is imperative to validate our results.

## 5. Conclusions

In this retrospective investigation concerning the HRQoL of PwH originating from Northern Greece, it was noted that individuals aged 46–60 years experienced the greatest impact, particularly in terms of physical activity. Predictably, those with severe haemophilia exhibited the lowest HRQoL levels. Subsequent studies are warranted to elucidate the disparities observed between the populations of Southern and Northern Greece, potentially facilitating the identification and amelioration of influential factors.

## Figures and Tables

**Table 1:** Demographic and clinical characteristics of the sample population (*n* = 100).

Demographic Characteristics	*n*	%
Age groups		
18–30 years	29	29.0
31–45 years	31	31.0
46–60 years	27	27.0
>60 years	13	13.0
Clinical Characteristics	*n*	%
Disease severity		
Severe	38	38.0
Moderate	18	18.0
Mild	44	44.0
Prophylaxis		
Yes	37	37.0
No	63	63.0
Comorbidities		
Yes	55	55.0
No	45	45.0
Hypertension		
Yes	20	20.0
No	80	80.0
Diabetes mellitus		
Yes	5	5.0
No	95	95.0
Hypercholesterolaemia		
Yes	12	12.0
No	88	88.0
Cardiopathy		
Yes	2	2.0
No	98	98.0
Psychiatric disorders		
Yes	2	2.0
No	98	98.0
Osteoporosis		
Yes	2	2.0
No	98	98.0
Thyroid		
Yes	1	1.0
No	99	99.0
Rheumatoid arthritis		
Yes	1	1.0
No	99	99.0
Chronic Obstructive Pulmonary Disease		
Yes	1	1.0
No	99	99.0
HIV		
Yes	6	6.0
No	94	94.0
HCV		
Yes	44	44.0
No	56	56.0

**Table 2:** Correlation between disease severity and prophylaxis treatment.

Disease Severity	Prophylaxis		
	Yes	No	*p*-Value
	*n* (%)	*n* (%)	
Severe	32 (84.2)	6 (15.8)	*p* < 0.001
Moderate	4 (22.2)	14 (77.8)	
Mild	1 (2.3)	43 (97.7)	

**Table 3:** Correlation between demographic and clinical characteristics.

Age Groups	Disease Severity			*p*-Value	Prophylaxis		*p*-Value
	Severe	Moderate	Mild		Yes	No	
	*n* (%)	*n* (%)	*n* (%)		*n* (%)	*n* (%)	
18–30 years	19 (65.5)	3 (10.3)	7 (24.0)	*p* < 0.001	20 (69.0)	9 (31.0)	*p* < 0.001
31–45 years	14 (45.2)	6 (19.3)	11 (35.5)		11 (35.5)	20 (64.5)	
46–60 years	5 (18.5)	8 (29.6)	14 (51.9)		6 (22.2)	21 (77.8)	
≥ 61 years	0 (0.0)	1 (7.7)	12 (92.3)		0 (0.0)	13 (100.0)	

**Table 4:** Descriptive statistical analysis of the questions Haem-A-QoL (*n* = 100).

Question	Mean Score ± SD	Frequency (%) of Replies Per Rating 1–5				
		1	2	3	4	5
Physical health	49.7 ± 24.95					
Swelling pain	3.09 ± 1.13	9	23	27	32	9
Joint pain	3.31 ± 1.15	9	14	28	35	14
Pain on movement	2.98 ± 1.19	9	27	29	22	12
Difficulty walking	3.03 ± 1.34	18	16	29	19	18
More prep time	2.53 ± 1.33	28	27	20	14	11
Mental dimension	46.38 ± 28.57					
Weight	2.97 ± 1.36	17	20	34	7	22
Anger	2.67 ± 1.36	28	18	25	17	12
Worry	3.38 ± 1.32	10	18	23	22	27
Exclusion	2.40 ± 1.32	35	20	24	12	9
Perception	44.00 ± 21.14					
Jealousy of healthy people	2.29 ± 1.25	38	16	33	5	8
Satisfied with my body	2.78 ± 1.28	12	18	25	26	19
Tough life	3.36 ± 1.22	7	17	33	19	24
I am different from others	2.81 ± 1.37	22	23	22	18 34	15
I do not think about haemophilia	2.56 ± 1.11	5	16	27		18
Sports/Leisure	58.45 ± 25.40					
Avoiding sports	3.76 ± 1.42	12	10	13	20	45
Avoiding football	3.67 ± 1.63	20	9	7	12	52
Sports like the others	3.36 ± 2.47	27	24	24	8	17
Lack of freedom of travel	2.47 ± 1.39	35	21	17	16	11
Planning ahead	3.43 ± 1.50	20	7	15	26	32
Work/Study	31.81 ± 24.79					
Normal work	1.85 ± 1.03	4	3	13	34	46
Normal studies	1.94 ± 1.12	5	6	11	34	44
Threat of daily activities	2.74 ± 1.39	26	18	28	12	16 5
Difficulty paying attention due to pain	2.56 ± 1.23	26	18	31	19	
Coping	24.33 ± 16.93					
Early recognition of bleeding	2.15 ± 1.11	3	12	16	35	34
I understand bleeding	2.06 ± 0.98	3	4	21	40	32
Bleeding control	2.44 ± 1.09	6	8	31	34	21
Treatment	41.59 ± 19.04					
Coagulation factor dependence	3.85 ± 1.22	5	11	19	24	41
Dependence on doctors	3.44 ± 1.34	10	17	21	23	29
Discomfort due to injections	2.18 ± 1.16	36	30	18	12	4
Interruption of everyday life	2.17 ± 1.15	37	27	21	12	3
Complications	2.72 ± 1.36	27	18	22	22	11
Problems with treatment	1.98 ± 1.19	49	21	19	5	6
Fear of ignorance of doctors	3.57 ± 1.35	11	13	16	28	32
Haemophilia Center Satisfaction	4.40 ± 0.55	0	0	3	34	63
Future	42.80 ± 23.68					
Difficulty living a normal life	3.00 ± 1.27	12	27 (27.0)	27 (27.0)	17 (17.0)	17 (17.0)
Improvement in the future	2.67 ± 1.23	9	18 (18.0)	23 (23.0)	31 (31.0)	19 (19.0)
Aggravation of the condition	2.66 ± 1.18	20	26 (26.0)	28 (28.0)	20 (20.0)	6 (6.0)
Influencing designs for life	3.30 ± 1.45	16	16 (16.0)	19 (19.0)	20 (20.0)	29 (29.0)
Need a wheelchair	1.97 ± 1.15	46	25 (25.0)	15 (15.0)	8 (8.0)	4 (4.0)
Family planning	15.68 ± 22.46					
Difficulty having children	1.41 ± 0.99	82	6	4	5	3
Fear of having children	1.40 ± 1.40	81	8	3	6	2
Fear of not taking care of children	2.06 ± 1.39	56	11	12	13	8
Non-family concern	1.64 ± 1.14	69	14	5	8	4
Relationships/Sexuality	24.16 ± 29.12					
Difficulty dating	1.93 ± 1.31	54	14	17	5	8
Gender insecurity	2.05 ± 1.25	45	23	17	7	7
Normal relationship inability	1.86 ± 1.26	56	15	13	9	5
Total score Haem-A-QoL	40.11 ± 17.38					

**Table 5:** Mean score of the 10 scales of the Haem-A-QoL questionnaire by age group.

	Age Group 18–30 Years		Age Group 31–45 Years		Age Group 46–60 Years		Age Group ≥61 Years	
	*n*	Mean Score ± SD (Min–Max)	*n*	Mean Score ± SD (Min–Max)	*n*	Mean Score ± SD (Min–Max)	*n*	Mean Score ± SD (Min–Max)
Physical health	29	35.00 ± 22.44 (0.00–85.00)	31	52.26 ± 23.12 (20.00–95.00)	27	61.48 ± 22.61 (0.00–100.00)	13	51.92 ± 25.70 (15.00–95.00)
Mental dimension	29	38.36 ± 30.05 (0.00–100.00)	31	45.56 ± 28.79 (0.00–100.00)	27	56.71 ± 26.51 (12.50–100.00)	13	44.71 ± 25.11 (0.00–87.50)
Perception	29	41.55 ± 19.04 (10.00–80.00)	31	45.16 ± 22.67 (10.00–95.00)	27	49.44 ± 21.18 (10.00–90.00)	13	35.38 ± 20.46 (5.00–80.00)
Sports/Free time	29	53.62 ± 27.45 (5.00–100.00)	31	60.16 ± 22.27 (20.00–95.00)	27	65.19 ± 22.55 (10.00–100.00)	13	51.15 ± 31.63 (0.00–100.00)
Work/Study	29	27.16 ± 21.99 (0.00–75.00)	31	35.08 ± 25.70 (0.00–93.75)	27	38.43 ± 27.39 (0.00–100.00)	13	20.67 ± 18.82 (0.00–50.00)
Coping	29	25.29 ± 16.17 (0.00–66.67)	31	23.44 ± 16.32 (0.00–60.00)	27	23.46 ± 16.98 (0.00–73.33)	13	26.15 ± 21.34 (0.00–80.00)
Treatment	29	38.15 ± 18.46 (3.13–84.38)	31	41.03 ± 20.01 (0.00–75.00)	27	45.83 ± 18.30 (12.50–78.13)	13	41.83 ± 19.95 (12.50–90.63)
Future	29	37.07 ± 24.69 (5.00–85.00)	31	43.55 ± 23.53 (0.00–95.00)	27	51.11 ± 19.63 (15.00–85.00)	13	36.54 ± 25.67 (0.00–85.00)
Family planning	29	16.81 ± 26.04 (0.00–75.00)	31	18.55 ± 20.44 (0.00–87.50)	27	16.20 ± 23.66 (0.00–8125)	13	5.29 ± 13.46 (0.00–43.75)
Relationship/Sexuality	28	21.13 ± 26.98 (0.00–100.00)	31	29.03 ± 31.69 (0.00–100.00)	27	29.01 ± 31.29 (0.00–100.00)	13	8.97 ± 16.48 (0.00–58.33)
Total score Haem-A-QoL	29	35.31 ± 17.41 (5.43–78.00)	31	41.43 ± 17.34 (14.13–84.24)	27	46.16 ± 16.01 (19.57–78.80)	13	35.16 ± 17.58 (13.59–73.91)

**Table 6:** Mean score of the 10 domains of the Haem-A-QoL questionnaire by condition severity.

	Severe		Moderate		Mild	
	*n*	Mean Score ± SD (Min–Max)	*n*	Mean Score ± SD (Min–Max)	*n*	Mean Score ± SD (Min–Max)
Physical health	38	49.34 ± 25.53 (0.00–100.00)	18	54.72 ± 21.73 (20.00–90.00)	44	47.95 ± 25.93 (0.00–100.00)
Mental dimension	38	45.72 ± 29.46 (0.00–100.00)	18	45.14 ± 22.84 (18.75–93.75)	44	47.44 ± 30.38 (0.00–100.00)
Perception	38	46.05 ± 20.27 (10.00–95.00)	18	41.11 ± 20.55 (10.00–70.00)	44	43.41 ± 22.38 (5.00–90.00)
Sports/Free time	38	63.68 ± 23.98 (15.00–100.00)	18	55.56 ± 22.81 (10.00–95.00)	44	55.11 ± 27.31 (0.00–100.00)
Work/Study	38	33.72 ± 27.24 (0.00–93.75)	18	29.17 ± 23.58 (0.00–81.25)	44	31.25 ± 23.46 (0.00–100.00)
Coping	38	22.63 ± 14.67 (0.00–66.67)	18	20.00 ± 11.66 (0.00–40.00)	44	27.58 ± 20.00 (0.00–80.00)
Treatment	38	40.13 ± 17.20 (0.00–84.38)	18	41.15 ± 20.37 (18.75–78.13)	44	43.04 ± 20.28 (3.13–90.63)
Future	38	42.89 ± 23.67 (0.00–95.00)	18	44.17 ± 22.64 (5.00–75.00)	44	42.16 ± 24.60 (0.00–85.00)
Family planning	38	20.89 ± 24.93 (0.00–87.50)	18	13.89 ± 20.06 (0.00–68.75)	44	11.93 ± 20.70 (0.00–81.25)
Relationships/Sexuality	37	27.93 ± 28.07 (0.00–100.00)	18	28.24 ± 31.07 (0.00–100.00)	44	19.32 ± 29.13 (0.00–100.00)
Total score Haem-A-QoL	38	41.23 ± 17.46 (14.13–84.24)	18	39.55 ± 16.11 (19.57–69.57)	44	39.38 ± 18.13 (5.43–78.80)

**Table 7:** Mean score of the 10 domains of the Haem-A-QoL questionnaire of patients on prophylaxis.

	*n*	Mean Score ± SD (Min–Max)
Physical health	37	48.51 ± 25.38 (0.00–100.00)
Mental dimension	37	44.09 ± 28.03 (0.00–100.00)
Perception	37	45.00 ± 17.79 (15.00–80.00)
Sports/Leisure	37	62.70 ± 23.29 (15.00–100.00)
Work/Study	37	31.25 ± 24.91 (0.00–93.75)
Coping	37	23.78 ± 15.91 (0.00–66.67)
Treatment	37	40.46 ± 16.71 (12.50–84.38)
Future	37	40.41 ± 23.61 (0.00–85.00)
Family planning	37	17.23 ± 22.98 (0.00–75.00)
Relationships/Sexuality	36	28.24 ± 28.96 (0.00–100.00)
Total score Haem-A-QoL	37	40.14 ± 16.34 (15.22–78.80)

## Data Availability

Data will be immediately available upon reasonable request.
